# Supplementary material for: Structure and Vibrational Spectroscopy of C82 Fullerenol Valent Isomers: An Experimental and Theoretical Joint Study
Source: Molecules. 2023 Feb 6;28(4):1569. doi: 10.3390/molecules28041569 (PMC9965979; doi:10.3390/molecules28041569)
Supplement: Supplementary file 1 [file molecules-28-01569-s001.zip › molecules-2155895-supplementary.pdf]

Supplement Materials

Structure and Vibrational Spectroscopy of Valent Isomers C_{82} Fullerenols: an Experimental and Theoretical Joint Study

Felix N. Tomilin[1,2,*], Polina V. Artyushenko[2,3,4], Irina A. Shchugoreva[3,4], Anastasia V. Rogova[2,4], Natalia G. Vnukova[1,2], Grigory N. Churilov[1,2], Nikolay P. Shestakov[1,2], Olga N. Tchaikovskaya[5], Sergei G. Ovchinnikov[1,2], Pavel V. Avramov[6,**]

[1]Kirensky Institute of Physics, Federal Research Center KSC SB RAS, Krasnoyarsk, Russia
[2]Siberian Federal University, Krasnoyarsk, Russia
[3]Laboratory for Digital Controlled Drugs and Theranostics, Federal Research Center KSC SB RAS, Krasnoyarsk, Russia
[4]Laboratory for Biomolecular and Medical Technologies, Krasnoyarsk State Medical University, Krasnoyarsk, Russia
[5]Department of Physics, Tomsk State University, Tomsk, Russia
[6]Kyungpook National University, Daegu, Republic of Korea

**e-mail: felixnt@gmail.com*
***e-mail: paul.veniaminovich@knu.ac.kr*

Abstract Gd@$C_{82}O_xH_y$ endohedral complexes for advanced biomedical applications (computer tomography, cancer treatment, etc.) were synthesized using high-frequency arc plasma discharge through a mixture of graphite and Gd_2O_3 oxide. The Gd@C_{82} endohedral complex was isolated by high-efficiency liquid chromatography and consequently oxidized with formation of a family of Gd endohedral fullerenols with gross formula Gd@$C_{82}O_8(OH)_{20}$. Fourier-transformed infra-red (FT-IR) spectroscopy was used to study structure and spectroscopic properties of the complexes in combination with DFTB3 electronic structure calculations and infra-red spectra simulations. It was shown that the main IR spectral features are formed by fullerenole C_{82} cage which allows one to consider the force constants at DFTB3 level of theory without consideration of gadolinium endohedral ions inside the carbon cage. Based on the comparison of experimental FT-IR and theoretical DFTB3 IR spectra it was found that oxidation of C_{82} cage causes formation of Gd@$C_{82}O_{28}H_{20}$ with breakdown of the integrity of parent C_{82} cage with formation of pores between neighboring carbonyl and carboxyl groups. The Gd@$C_{82}O_6(OOH)_2(OH)_{18}$ endohedral complex with epoxy, carbonyl and carboxyl groups was considered as the most reliable fullerenole structural model.

Keywords: C_{82}, Gd endohedral complexes, biomedical applications, fullerenols, DFTB3 electronic structure calculations, IR spectra

CARTESIAN COORDINATES

Atomic Cartesian coordinates for equilibrium structures of studied complexes of C_{82} fullerenols (in Å: X, Y, Z) are presented below.

$C_{82}O_3(OH)_{24}$

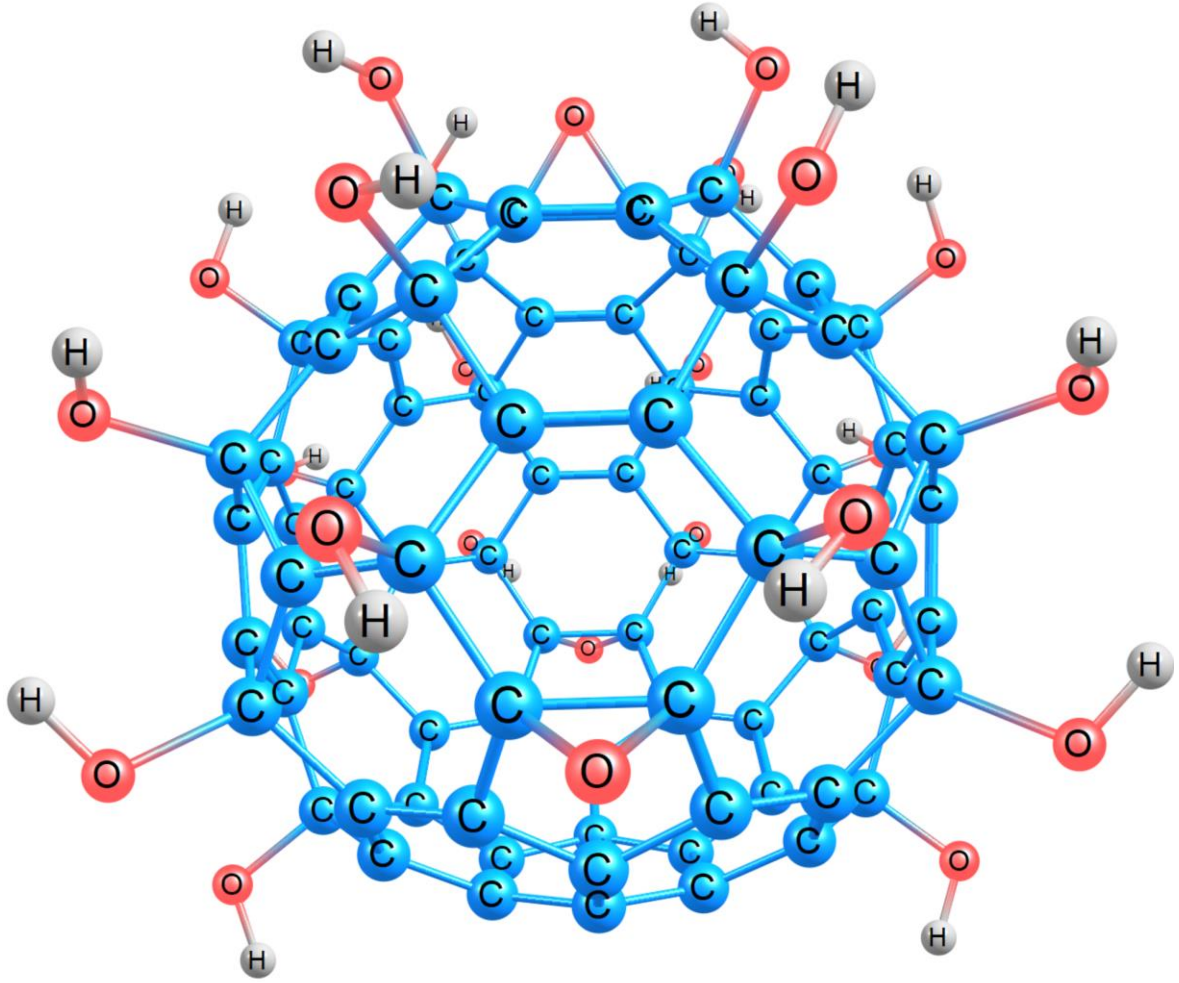

DFTB3/3ob-3-1// Ground state

C	-2.633905408817	0.397581121835	3.485446169396
C	2.682739063779	0.283566937873	3.441110737430
C	-3.069223003628	1.505346063341	2.695103838800
C	3.103964922613	-0.970587089549	2.901719552697
C	3.149631432585	1.371667828387	2.641785713787
C	-3.115135761575	-0.837255108604	2.952054986657
C	-3.955905427232	0.975910383540	1.601788165265
C	3.966350850176	-0.693715625664	1.701098095242

C	3.993662188175	0.804644688643	1.534591913286
C	-3.983401953190	-0.522681295738	1.765919305216
C	-2.502867795853	3.603899445348	1.418396695341
C	2.515638565872	-3.295234299488	2.124224199043
C	2.655895898788	3.494794607427	1.374880881357
C	-2.643150805825	-3.186209759558	2.166846781993
C	-4.020432878162	1.648186122234	0.217475130883
C	4.003065669160	-1.651135169256	0.495013879765
C	4.056954973173	1.468279758319	0.149852897939
C	-4.074573712134	-1.471523237340	0.560342533732
C	-3.129461701560	2.892635708688	0.201950261893
C	3.114847825597	-2.867056546687	0.769279761662
C	3.236088726545	2.761431789761	0.147777842919
C	-3.250914524502	-2.736017647769	0.822147546623
C	-2.315100314935	2.677981455769	2.617907541834
C	2.351004969954	-2.130127814047	3.097263711604
C	2.446336569873	2.575980054844	2.576656711835
C	-2.410444144881	-2.028496948059	3.135350759586
C	-3.637159688357	0.615714910697	-0.924200797605
C	3.593348941349	-0.890688044602	-0.835546455646
C	3.604861826355	0.456004250779	-0.977823542549
C	-3.648531627321	-0.729654828654	-0.769869735662
C	-2.507477701867	3.465335530430	-1.064268879539
C	2.467553859862	-3.699931552329	-0.328666436856
C	2.611806855824	3.359523425496	-1.110084883511
C	-2.651765592782	-3.594673858356	-0.288437871890
C	-2.337364891945	2.421128584912	-2.142763693029
C	2.272336351965	-2.914911918655	-1.604646154271
C	2.373734533901	2.318929129929	-2.176154430988
C	-2.438633500916	-2.811397019732	-1.560078648290
C	-3.317743554511	1.254691565407	-2.308851580954

C	3.246239497512	-1.814747228184	-2.041001084078
C	3.303154311485	1.121794829468	-2.354834080917
C	-3.374401702490	-1.679665667236	-1.975214789107
C	-2.485066071897	0.325880629849	-3.200713192524
C	2.392772299914	-1.100184294477	-3.095732192581
C	2.426450281899	0.226521144910	-3.247058245526
C	-2.518842808835	-1.001892303563	-3.059105559609
C	-1.232397923443	2.308853057940	-2.904624047705
C	1.151367177490	-2.968609695641	-2.349332943946
C	1.249628982441	2.249495030968	-2.913901673671
C	-1.330512890385	-2.906675445698	-2.318820439939
C	-1.340899512371	1.114622201489	-3.873109125257
C	1.236560114441	-2.013709068086	-3.556214140365
C	1.303932869417	1.056809672497	-3.891608802253
C	-1.408288075353	-1.955119134112	-3.531242225397
C	-1.130440384485	3.888621236260	-0.570200568766
C	1.102203483515	-4.002229788186	0.274552960871
C	1.262247192406	3.838843542262	-0.590747394727
C	-1.290460376392	-3.952713840231	0.294135098876
C	-1.119965488484	3.968990300197	0.832205625633
C	1.121541548515	-3.775971086298	1.660816509255
C	1.279755281409	3.918760105199	0.811286916657
C	-1.278179005421	-3.725661618325	1.680012684213
C	-1.124918822489	2.766869634752	3.393046850471
C	1.177778441452	-2.045644228086	3.898312567231
C	1.275175174424	2.715841487770	3.372959907480
C	-1.222291757461	-1.994464633116	3.917905349213
C	-0.673313444695	1.667906926268	4.139037428110
C	0.739436637663	-0.809668337657	4.396734492998
C	0.789597247653	1.636582209268	4.126499167143
C	-0.723486802670	-0.778361716667	4.408472699984

C	-1.418930768379	0.453298277781	4.217070697108
C	1.483633947326	0.391126487844	4.192844464111
C	-0.034711159976	0.275542123857	-4.013125622198
C	-0.074277821943	-1.222167102457	-3.848027876236
C	0.029012952000	3.190477054578	-2.750615231740
C	-0.104585555945	-3.792567929274	-1.980607209098
C	0.056858948995	3.765024514295	-1.314150829384
C	-0.100776029938	-4.040738157145	-0.453279597771
C	0.086296266964	3.961466479185	1.548201606308
C	-0.069366761950	-3.610100015363	2.383321760943
C	0.092223217944	3.671476844332	3.042383558594
C	-0.044451607962	-3.001954366657	3.778407267272
O	0.044845563967	4.229416084080	-3.725089757322
O	-0.138721641912	-5.018569271745	-2.705130241789
H	-0.115076781933	3.844835044240	-4.603132077916
H	0.000426979999	-4.834460939795	-3.649175131356
O	0.124258940936	4.887671408777	3.780797993297
O	-0.057963056952	-4.028371857194	4.764160904815
H	0.127556064930	4.693150019898	4.731275862869
H	-0.042018624965	-3.631745184370	5.649427457432
O	-3.266047285533	4.763805810847	1.789422401204
O	3.289112665521	-4.348456250041	2.722493337761
O	3.472570283449	4.620733582897	1.737087861224
O	-3.449292355450	-4.204533180112	2.782463170734
H	-3.389103928457	5.318418676592	0.999678596536
H	3.397048576459	-5.061739474723	2.069795438037
H	3.612367023345	5.168025826667	0.944973015555
H	-3.605019316384	-4.910413564761	2.131308854057
O	-5.156061987657	0.287478647848	2.047394819081
O	5.173869583628	0.072554103943	1.960881932087
O	-0.067399225956	-0.607746269746	-5.153090329685

O	-3.282172160507	4.595289423908	-1.545522114291
O	3.234550363514	-4.909295644749	-0.568870425764
O	3.416775313470	4.451034774002	-1.622043502246
O	-3.464811814449	-4.769562040832	-0.533583126757
H	-4.166341508126	4.269840673074	-1.789971228180
H	4.112710121135	-4.646892108894	-0.896566057587
H	4.217731319075	4.070265773179	-2.022984033085
H	-4.275031140084	-4.483116005972	-0.990754276554
O	-4.549449001950	1.655257964226	-2.914971534652
O	4.465867103964	-2.340332175950	-2.571323143814
O	4.533540786960	1.524647727297	-2.964669174663
O	-4.616398299925	-2.202680350991	-2.456772592883
H	-4.360055551015	1.995310743086	-3.805576222248
H	4.258499414061	-2.865837246686	-3.362230816478
H	5.232954001620	0.890505517610	-2.735118223773
H	-5.311930248601	-1.531623052280	-2.357402374907
O	-5.381453673581	2.068529205041	-0.034210528982
O	5.359314780560	-2.119256723040	0.311966234874
O	5.442233013546	1.800439851208	-0.148206193910
O	-5.464985213526	-1.857177503146	0.371328159818
H	-5.968603256275	1.543427662279	0.540671823733
H	5.957695387291	-1.479995123316	0.741471184642
H	6.000580798292	1.426255337329	0.558711820751
H	-6.009759731272	-1.329380009421	0.984690153571
O	-1.693288588208	1.591536863269	-5.177007078660
O	1.562593857282	-2.763325480763	-4.732418663843
O	1.640688863274	1.522515216327	-5.203628739633
O	-1.771797758212	-2.694198722762	-4.703070205847
H	-1.389861872395	0.928641916556	-5.825875350362
H	1.249381698442	-2.253435908982	-5.503516440508
H	2.517948371858	1.939493477124	-5.171804406653

H	-2.647407990807	-3.091830970581	-4.562820263950

First isomer

$C_{82}O_8(OH)_{20}$

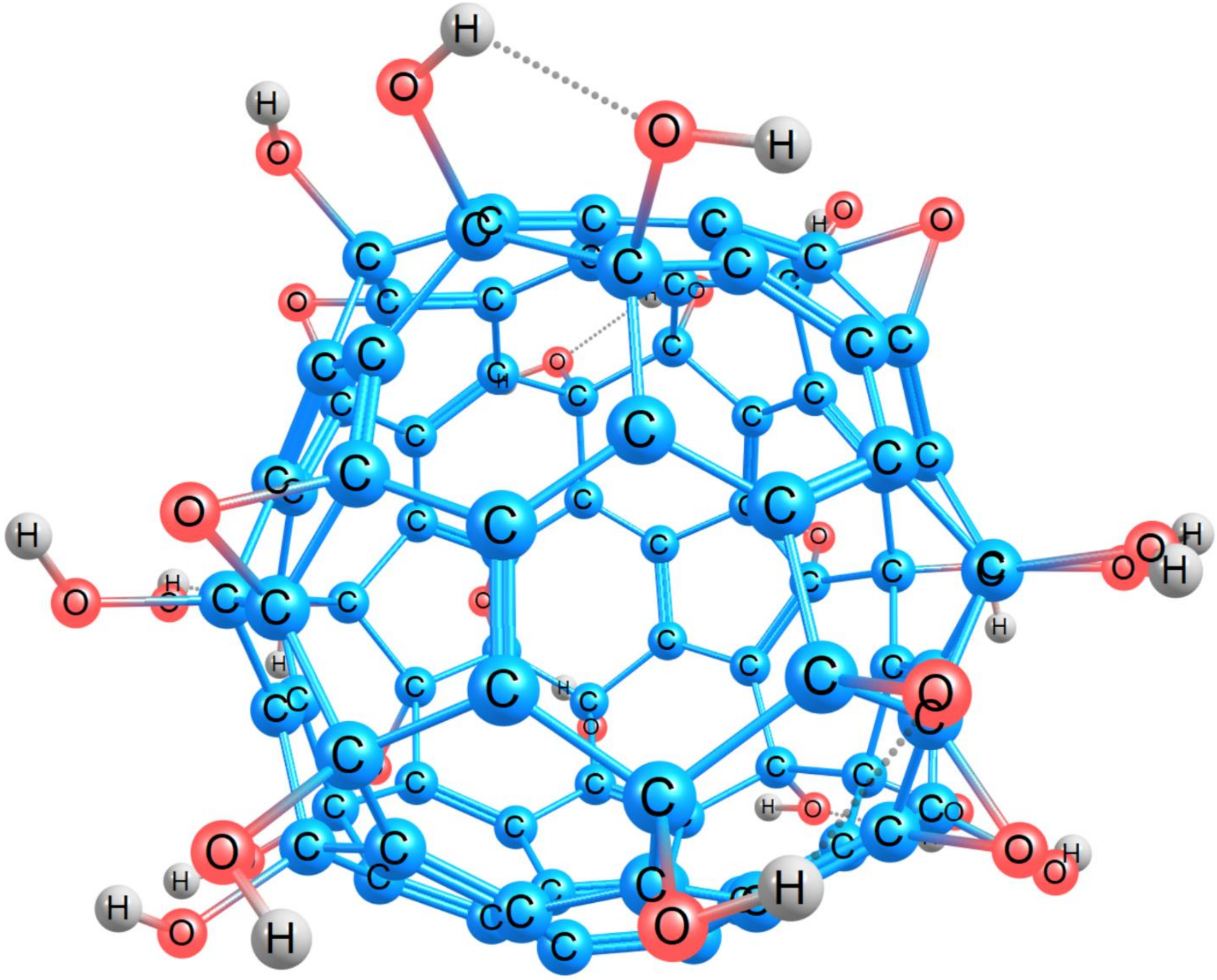

DFTB3/3ob-3-1// Ground state

C	-2.765254908745	-0.043666591981	3.117980122574
C	2.726886020748	-0.055216278990	3.062574343623
C	-3.355090136464	1.211616035466	2.465873579907
C	3.379210082464	1.191329363467	2.444209692895
C	-3.425757084427	-1.289839417424	2.486555171860
C	3.399060264462	-1.294617994400	2.431489772893
C	-3.743010316284	0.630278337705	1.076692924528
C	3.680921512346	0.644564036724	1.019519707536
C	-3.752357311301	-0.716332028655	1.071837746500
C	3.662168988322	-0.701351440690	1.006677973525
C	2.614036759803	3.351843261500	1.190793347448

C	-2.607218414827	3.445541591454	1.267236194439
C	2.347643416936	-3.215130115541	1.060609799523
C	-2.549218681870	-3.386073562449	1.169328674451
C	4.093279255167	1.494089822315	-0.198229030909
C	-4.056586869171	1.495048473310	-0.141879742947
C	4.004473681177	-1.527965935300	-0.234462225888
C	-4.123872027108	-1.545586647298	-0.169371561927
C	3.422736060461	3.012219939612	-0.132233451945
C	-3.292792370485	2.868259799706	-0.034340843959
C	3.213434266556	-2.873073730696	-0.133383907944
C	-3.433173985441	-3.067724579615	-0.093615598965
C	-2.344599715926	2.359325112924	2.308355496968
C	2.479517621877	2.453437034904	2.393707528890
C	-2.508886908872	-2.549307457862	2.418901616915
C	2.547153347835	-2.659620095811	2.475719107889
C	-3.665785220327	0.669127667684	-1.432817416368
C	3.730602930323	0.671843258691	-1.518283222298
C	-3.744955725312	-0.703187025702	-1.454972976356
C	3.643285348358	-0.701806389665	-1.514996210299
C	-2.487106484858	3.377448403459	-1.186171653482
C	2.375842328906	3.259177251529	-1.210864263459
C	-2.445716007894	-3.351951557465	-1.229512142418
C	2.476475713878	-3.434849522433	-1.317193663390
C	2.369051468920	2.623516611807	-2.416797961928
C	-2.393163734918	2.581031862809	-2.413133485892
C	2.418476282922	-2.637111239787	-2.545403271835
C	-2.383564927943	-2.651150719776	-2.380467976938
C	-3.019622509606	1.384902354372	-2.547481871867
C	3.218093766532	1.433039629326	-2.709508380763
C	-3.228724865550	-1.451138873346	-2.652609468778
C	3.011335008616	-1.416019103337	-2.643376747788
C	-2.596815719823	0.757815603667	-3.901182578245
C	2.502210375884	0.738597147648	-3.848790371280
C	-2.529364634867	-0.747113653637	-3.790904420268

C	2.566551618813	-0.766803847678	-3.979993308213
C	-1.361679796358	2.857813651682	-3.528603462407
C	1.185035348476	2.597285994829	-3.239756602545
C	-1.206079859448	-2.616441240837	-3.238651984515
C	1.342349324416	-2.886833653699	-3.634583269326
C	-1.224685459420	1.411731498335	-4.085353182133
C	1.191747392485	1.456152738350	-4.015325001169
C	-1.226470325437	-1.466998791335	-3.992668037165
C	1.190297414437	-1.423568020368	-4.150478684107
C	-1.227054859442	3.977828602205	-0.645499282686
C	1.181182211490	3.902443822210	-0.745245759656
C	-1.257241159437	-4.123515076151	-0.785301099667
C	1.183750382447	-3.973119877208	-0.807922735639
C	-1.257042399404	4.023919706168	0.719986178696
C	1.337087760380	4.381491013005	0.704475164678
C	-1.289625678394	-4.156519938114	0.722853939648
C	1.230927603451	-3.891167216221	0.662688388723
C	1.130832429471	2.388747528892	3.007064336652
C	-1.160737707454	2.338995962914	2.979744759628
C	1.152742635475	-2.465154370874	3.063498569628
C	-1.171824855447	-2.470889732861	3.078219776601
C	-0.704536844678	1.148218475490	3.685600735341
C	0.681828713680	1.160192544449	3.657001800349
C	-0.740967175645	-1.296962465412	3.701644846331
C	0.721858691654	-1.289164782420	3.690305751335
C	1.470003833354	-0.059857093983	3.632991059335
C	-1.492333735337	-0.067747192965	3.659121100356
C	-0.052018961000	0.742710455686	-4.308400144046
C	0.012535478976	-0.753304737673	-4.323878801045
C	-0.006958270977	3.512842276404	-2.962843769646
C	-0.015167437011	-3.559573464383	-3.051097231634
C	-0.008423267022	3.885498111248	-1.438339268353
C	0.036942992964	-3.966771824225	-1.537100990279
C	-0.022017302006	4.272111075041	1.629324211239

C	0.025532051997	-4.230198174081	1.567170735281
C	0.028146436003	3.113355899563	2.612356090796
C	-0.002020827000	-3.186540699548	2.662668210794
O	-5.455063287536	1.775070760191	-0.160195208951
O	-3.771361101278	3.948711740220	-0.842141231596
O	4.491023293955	3.940853103206	-0.258983729894
O	5.493677521526	1.643239689229	-0.128222422967
O	5.407606980568	-1.777623994168	-0.265310485875
O	3.698045094334	-4.027423123192	-0.837791287609
O	-5.526882859485	-1.726351892241	-0.165660655932
O	-4.500869415939	-4.012148478204	-0.115944364963
O	-3.763823844296	-1.450399126363	-3.989006929184
O	-3.487625231400	1.147089198488	-4.944026396772
O	3.310683046490	-3.599428815346	3.262154008495
O	4.685683400882	-1.520298316297	3.015495858627
O	-1.940704558103	3.699570112309	-4.522720974974
O	0.176494306908	4.749327252862	-3.691456127322
O	3.442370974425	-1.133799293469	-5.041998067701
O	3.735990202320	1.442594771346	-4.052443095181
O	-0.241207162866	-4.752515274849	-3.827957416271
O	1.881475914121	-3.706249128302	-4.665244621909
O	-1.559571429303	-5.339298637570	-0.044089924996
O	0.149099091933	-5.552638559462	2.105022177047
O	-3.210430526524	-3.812402129273	2.356629768927
O	-4.628058466919	-1.650240780276	3.156349573562
O	-4.522812583970	1.676839813259	3.154985751550
O	-3.449532979436	4.448393140966	1.838299524161
O	-0.120076516951	5.504208243504	2.320569823934
O	1.840420209184	5.609157087463	0.767357490644
O	4.584990429917	1.528755903321	3.118063361573
O	3.241555313533	3.692587806313	2.355927411914
H	1.276370288432	-4.456531058999	-4.826447183837
H	-1.111175842472	-5.125277006686	-3.604896260342
H	-1.368067719354	4.481973615958	-4.648398208888

H	1.015583234553	5.159390650669	-3.420076285430
H	-3.411023235462	2.109868667043	-5.072679550705
H	3.395871618470	-2.099140253071	-5.163701011668
H	-2.905339050678	5.065294246706	2.356668119937
H	-4.880740857798	2.454227821880	2.690822227790
H	4.652446279903	-2.364756249905	3.510575809400
H	2.796024005720	-4.420439137995	3.354308488484
H	-5.213099695638	-0.874641943588	3.197860213556
H	5.171189602652	0.750794949678	3.116770415604
H	5.746451318408	2.535741703856	-0.430062609824
H	4.157289241128	4.834190797826	-0.053870312000
H	-4.646801186877	-4.332002253012	0.794543182649
H	-5.724440326417	-2.650440855802	-0.423336672795
H	5.701971800421	-2.091722501034	0.605208824725
H	-5.614987267429	2.501170866870	-0.788482636639
H	-0.258910439903	-6.175787850214	1.475925596333
H	0.599570082725	6.080078922257	1.957854670127

Second isomer

$C_{82}\,O_6\,(OOH)_2\,(OH)_{18}$

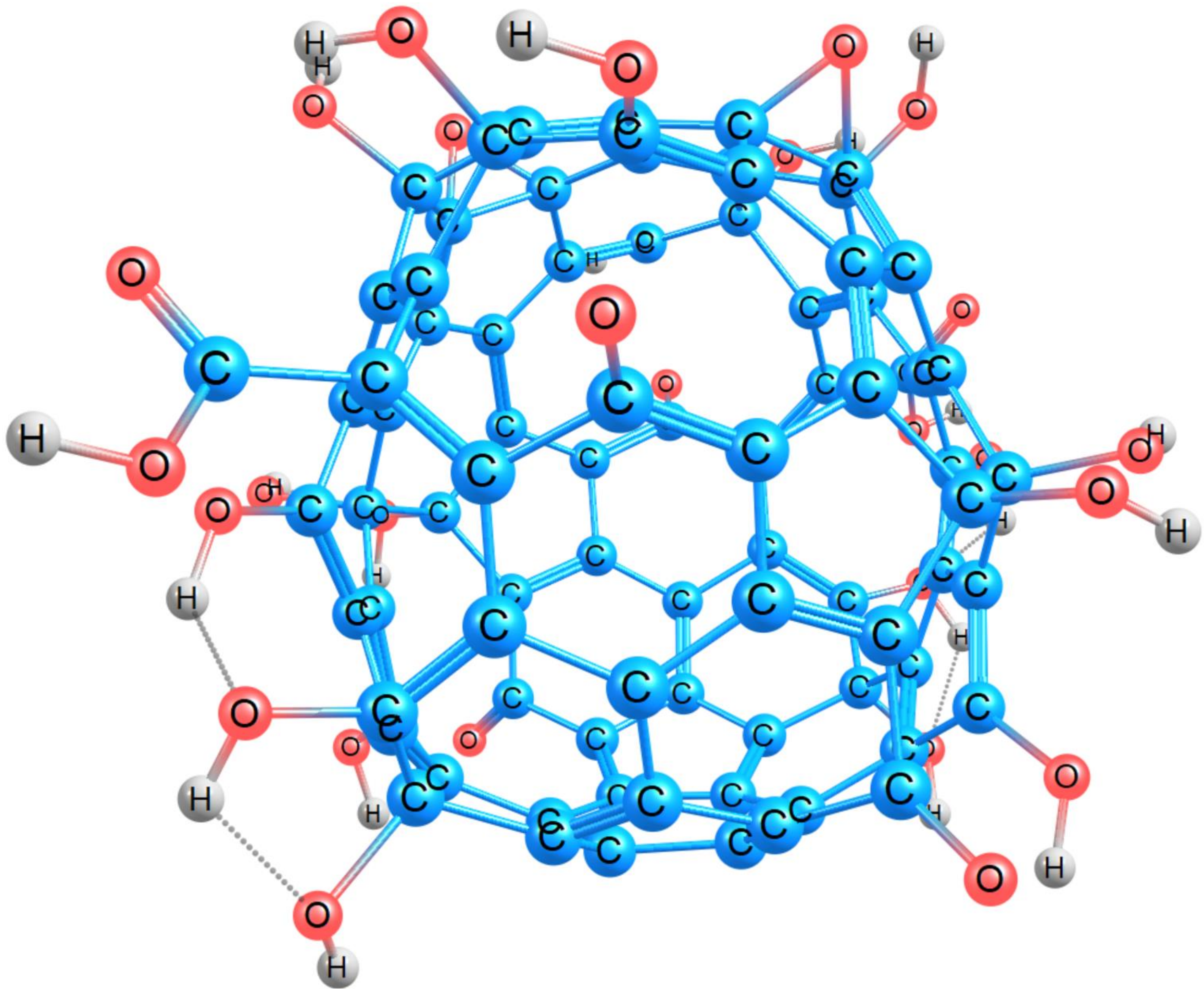

DFTB3/3ob-3-1// Ground state

C	-2.815265689710	-0.004010644001	3.169549066584
C	2.842191235693	-0.032626075988	3.147448682585
C	-3.142173369572	1.146448036484	2.346297920914
C	3.523276714423	1.092677568483	2.476644576878
C	-3.537556951380	-1.111056647519	2.498733696863
C	3.164417208548	-1.168577408495	2.306297255973
C	-3.554642873410	0.644053804708	1.072738636528
C	3.639858459359	0.798993114624	1.117756752495
C	-3.671564438354	-0.793670358647	1.142213250463
C	3.552168942413	-0.646577850696	1.033423248557
C	2.161086188041	3.363489201458	1.306551419424

C	-2.723566331789	3.597832593345	1.749566194190
C	2.744949734750	-3.616755855350	1.678335749240
C	-2.138882508008	-3.322112884483	1.278923634435
C	3.818704707251	1.668764689251	-0.127950298965
C	-3.890423402246	1.243517234457	-0.175527807896
C	3.869962669244	-1.230636983433	-0.222678376888
C	-3.855794256277	-1.648083295262	-0.114467438934
C	3.095875475576	3.140755361555	0.019454645976
C	-3.952953388188	4.348447608047	-1.566378074276
C	3.944752805197	-4.325995081037	-1.647863879272
C	-3.078691709625	-3.110300377580	0.041525218986
C	-2.677512495769	2.526615261872	2.828095650692
C	2.211134601976	3.354410329493	2.823919504707
C	-2.200732027995	-3.322767165478	2.780900015749
C	2.707532844774	-2.556864822838	2.773494589732
C	-3.476449365413	0.597562138704	-1.401543698349
C	3.340184722467	0.860620440598	-1.395893211366
C	-3.365022184450	-0.829527410627	-1.373653725402
C	3.455871464419	-0.569167946729	-1.438956228329
C	-2.627434636825	3.704050835309	-1.438955835362
C	2.073464049064	3.455706039430	-1.118625723505
C	-2.096572694055	-3.426553803427	-1.133927650490
C	2.611653833798	-3.688922330344	-1.520683567336
C	2.115419993036	2.817082604713	-2.413440517919
C	-2.321549503957	2.670274977800	-2.484951925878
C	2.301674185934	-2.639528619782	-2.547122204855
C	-2.146002141003	-2.771773639753	-2.420229057885
C	-2.897393776712	1.410075973345	-2.502796059844
C	2.855744455712	1.581836883266	-2.511861460862
C	-2.889050902668	-1.539422412287	-2.503294071887
C	2.868556618717	-1.374321666367	-2.547309130844
C	-2.595415580800	0.707094421665	-3.835173818269
C	2.391077812926	0.840746495631	-3.706144575313
C	-2.433686036915	-0.785811710626	-3.694487188299

C	2.548240259860	-0.649548346701	-3.864061527243
C	-1.442160504365	2.872706414692	-3.842403255278
C	1.056171749535	2.822870221736	-3.381978669461
C	-1.084350116517	-2.768513630769	-3.398198234459
C	1.403130590374	-2.815363857699	-3.916588041246
C	-1.295657200408	1.401281380372	-4.254007880076
C	1.141788708490	1.540413611282	-4.166686452104
C	-1.188178025476	-1.478329087330	-4.171686351114
C	1.241637993434	-1.331841751403	-4.271856244074
C	-1.623411932281	4.085174052141	-0.499754056783
C	0.931835249577	3.994704958192	-0.569657256717
C	-0.933994351598	-3.951101998187	-0.604531771751
C	1.622536616277	-4.086251871155	-0.573304206720
C	-1.610101236258	3.944899458205	0.945420224571
C	0.928217844598	3.806930110280	0.860604501620
C	-0.902635887589	-3.761429067314	0.817503440624
C	1.629782532237	-3.951386731213	0.875859357602
C	1.021889217522	2.547928820845	3.313440315497
C	-1.288874405401	2.334260364921	3.361347138469
C	1.324627545384	-2.372300326923	3.328510577467
C	-0.986510412550	-2.582601562817	3.291646090488
C	-0.751467347638	1.138731408472	3.861341217246
C	0.731584313681	1.258144934435	3.818890984257
C	-0.694047026673	-1.299232110390	3.815983942266
C	0.789394189642	-1.182015032444	3.851226746251
C	1.541273943314	0.049652530984	3.793571477255
C	-1.506730077328	-0.094463376955	3.799111497270
C	-0.095929435958	0.775841542669	-4.441316223000
C	0.042815047996	-0.704628709690	-4.450729185994
C	-0.101654526932	3.585161747389	-3.675891868336
C	0.073551655976	-3.528393763381	-3.711238660306
C	-0.312779725882	4.586886147932	-1.083705703513
C	0.290717829844	-4.572526782943	-1.126178914489
C	-0.303905257837	3.803529666280	1.618776403240

C	0.326545370826	-3.801545923281	1.560965066273
C	-0.224058253873	3.129387514561	2.861818549718
C	0.255726206879	-3.151401210595	2.818546995697
O	-4.605168884905	2.347909522923	-0.243710704876
O	-4.209147462105	5.360237231546	-0.689778195663
O	4.088263120152	4.135247253109	-0.014303483978
O	5.216521478658	1.965501771129	-0.352376352828
O	4.578518152945	-2.342803318924	-0.310036413872
O	4.171364383112	-5.373671957561	-0.805321111631
O	-5.233470362620	-1.924324507139	-0.361931343821
O	-4.093821390168	-4.123269909126	0.061138779960
O	-2.388644669905	-1.469161702321	-4.977638000721
O	-3.659313413317	1.000191439565	-4.763865048853
O	3.680735138342	-3.026146318652	3.786078860290
O	-2.221703475993	3.656795360367	-4.742063677834
O	-0.075944920940	4.856704703787	-3.951682643215
O	3.598139100375	-0.930294958595	-4.814628785807
O	2.334086519928	1.532364280325	-4.983488221741
O	0.085328969954	-4.812697139832	-3.949337047226
O	2.176340570038	-3.526277067402	-4.859478806822
O	-2.949290407649	-4.001687053204	3.474467317422
O	-4.287499246064	-2.028505825060	3.117681400588
O	4.211666117099	2.055111324061	3.096977703578
O	2.878753579714	4.088424890166	3.531846460406
H	3.049545295630	-3.097994723617	-4.935907754761
H	-3.118588198563	3.271169647536	-4.794668156844
H	5.698411328405	1.135923403485	-0.504178438757
H	4.897012673758	3.803051126266	0.411225344837
H	-4.273571811050	-4.379041940003	0.982293081547
H	-5.343488712559	-2.895453688667	-0.445724471797
O	4.758029316865	-4.074636446150	-2.542625044869
O	3.950580606194	-4.160236550121	1.518932960306
O	-4.732869414854	4.141906186138	-2.503636534870
O	-3.924670362224	4.141686800098	1.602346243265

H	-4.867720425815	2.680262031803	0.629817348702
H	4.815862629822	-2.709125635749	0.557970903757
H	-5.027024266700	5.805561412345	-0.966669595561
H	4.994039740755	-5.813382636369	-1.076613975516
H	-3.690626260342	0.276151542898	-5.415695940551
H	3.604114234372	-0.206756036886	-5.468317921516
H	4.019249315194	2.104444660038	4.045311088169
H	-4.059030317177	-2.135289299024	4.053713177147
O	-3.641567149360	2.984058054650	3.853387144226
H	-4.440942948989	3.935155040225	2.411993516916
H	-3.612908069351	2.359442398923	4.599688890894
H	4.462705265985	-3.976884550205	2.336345548923
H	3.627531843363	-2.434944160905	4.557655609942
O	0.297754584845	-5.631045318428	-1.746059830230
O	-0.376674590815	5.621635938440	-1.736844887215
H	-0.970886692562	5.162910590671	-4.196958448121
H	-0.758715535673	-5.247276914605	-3.748203558298
